# 497. Moderate Versus High Dose Corticosteroids in Adult Patients with Severe COVID-19: Less Is More

**DOI:** 10.1093/ofid/ofab466.696

**Published:** 2021-12-04

**Authors:** Seema Joshi, Sana Soman, Saniya Jain, Atheel Yako, Marwa Hojeij, Louis Massoud, Ayman Alsaadi, Zachary R Smith, George J Alangaden, Rachel Kenney, Ramesh Mayur

**Affiliations:** 1 Henry Ford Hospital, Detroit, Michigan; 2 Henry Ford Health System, Grosse Pointe Park, Michigan

## Abstract

**Background:**

The early administration of corticosteroids (CS) in patients with severe COVID-19 (hospitalized with need for supplemental oxygen) has been the only therapy to improve survival. However, the optimal dosing of CS remains unclear. Beginning March 2020 methylprednisolone (MP) in a dose of 40mg twice daily (high dose CS - HDC) was adopted at our institution. Based on emerging trials, this dose of MP was reduced to 16mg twice daily (moderate dose CS – MDC) in November 2020. The study aims to evaluate the outcome difference in patients receiving HDC versus MDC.

**Methods:**

This pre-post quasi-experimental study was done at Henry Ford Hospital, an 877-bed tertiary care hospital in Detroit, Michigan. Consecutive patients in the HDC group from September 1, 2020 to November 15, 2020 were compared to the MDC group from November 30, 2020 to January 20, 2021. Only hospitalized patients with severe COVID-19 were included. The primary outcome was 28-day mortality. Secondary outcomes included progression to mechanical ventilation, length of hospital stay, discharge on supplemental oxygen and CS-associated adverse events. Patient demographics were evaluated using descriptive statistics. Bivariate and multivariable logistic regression analysis was planned to test the association between primary outcome and exposure.

**Results:**

470 patients were evaluated, 218 and 252 in the HDC and MDC groups respectively. Clinical characteristics and severity of illness on admission were comparable in both groups (Table 1). Among comorbidities - lung disease, cardiovascular disease and hypertension were higher in MDC. Antibiotic and tocilizumab use were lower in MDC. Significantly more patients in MDC group received oral CS. There was no difference in mortality between HDC and MDC through bivariate and multivariate analysis (14.7% and 13.5%, p < 0.712, adjusted OR 0.913 [0.514-1.619]) (Table 2,3). Median length of hospital stay was 5 and 6 days in HDC and MDC respectively (p < 0.001). There was no difference in CS-associated adverse events.

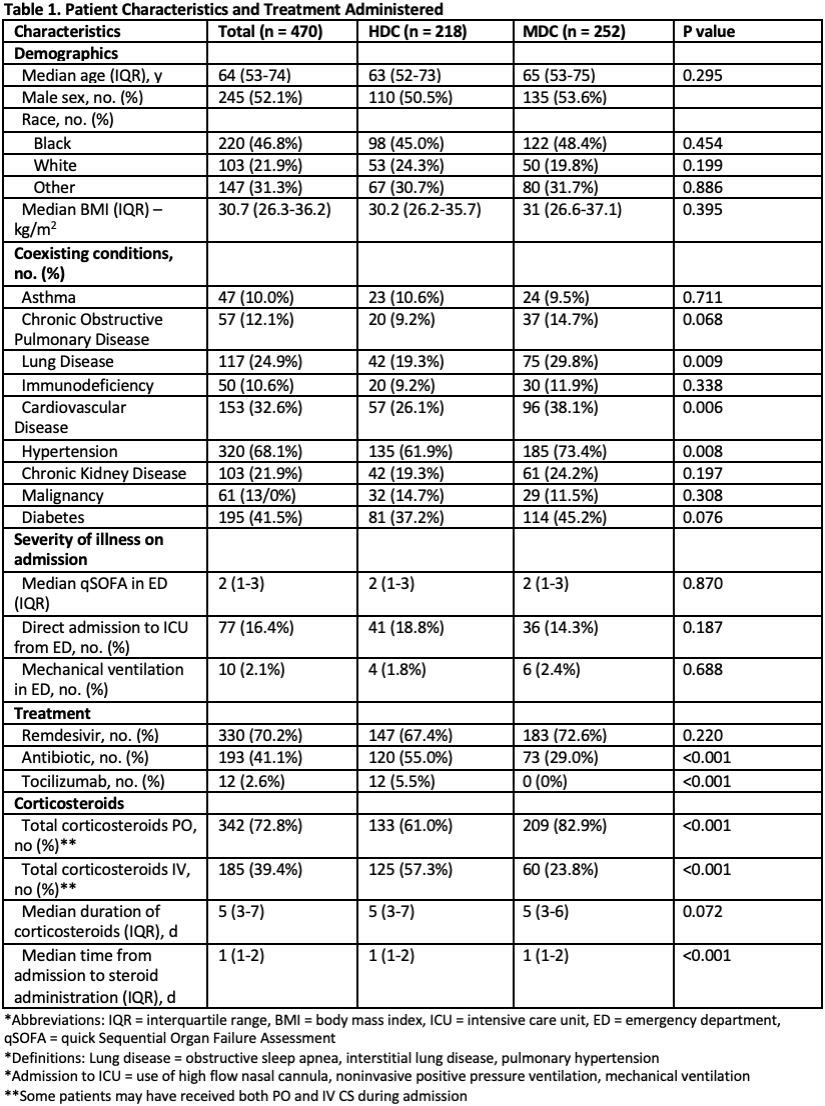

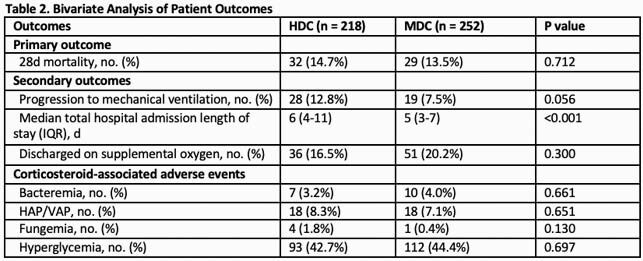

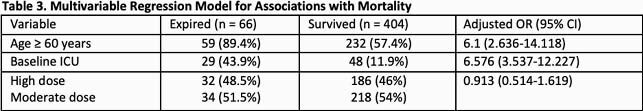

**Conclusion:**

The survival in severe COVID-19 patients treated with MDC is comparable to HDC. Oral corticosteroids are an equally effective option.

**Disclosures:**

**Rachel Kenney, PharmD**, **Medtronic, Inc.** (Other Financial or Material Support, spouse is an employee and shareholder)

